# Effect of HIIT with Tabata Protocol on Serum Irisin, Physical Performance, and Body Composition in Men

**DOI:** 10.3390/ijerph17103589

**Published:** 2020-05-20

**Authors:** Eugenia Murawska-Cialowicz, Pawel Wolanski, Jolanta Zuwala-Jagiello, Yuri Feito, Miroslav Petr, Jakub Kokstejn, Petr Stastny, Dawid Goliński

**Affiliations:** 1 Department of Physiology and Biochemistry, University School of Physical Education, 51-612 Wroclaw, Poland; pawel.wolanski@onet.pl (P.W.); dawidgolinski@o2.pl (D.G.); 2 Department of Pharmaceutical Biochemistry, Medical University, 50-556 Wroclaw, Poland; jolanta.zuwala-jagiello@umed.wroc.pl; 3 Department of Exercise Science and Sport Management, Kennesaw State University, Kennesaw, GA 30144, USA; yfeito@kennesaw.edu; 4 Faculty of Physical Education and Sport, Charles University, 162-52 Prague, Czech Republic; petr@ftvs.cuni.cz (M.P.); kokstejn@ftvs.cuni.cz (J.K.)

**Keywords:** HIIT, power output, fat reduction, fitness, lean body mass, irisin, health, basal metabolism

## Abstract

High-intensity interval training (HIIT) is frequently utilized as a method to reduce body mass. Its intensity of work results in a number of beneficial adaptive changes in a relatively short period of time. Irisin is a myokine and adipokine secreted to the blood during exercise and it takes part in the regulation of energy metabolism. It is a vital issue from the prophylaxis point of view as well as treatment through exercise of different diseases (e.g., obesity, type-2 diabetes). The aim of this study was to evaluate changes in irisin concentration, body composition, and aerobic and anaerobic performance in men after HIIT. Eight weeks of HIIT following the Tabata protocol was applied in the training group (HT) (*n* = 15), while a sedentary group (SED) (*n* = 10) did not participate in fitness activities within the same time period. Changes of irisin, body composition, and aerobic and anaerobic performance were evaluated after graded exercise test (GXT) and Wingate anaerobic test (WAnT) before and after eight weeks of training. Training resulted in an increased of blood irisin concentration (by 29.7%) *p* < 0.05), VO_2max_ increase (PRE: 44.86 ± 5.74 mL·kg^−1^·min^−1^; POST: 50.16 ± 5.80 mL kg^−1^·min^−1^; *p* < 0.05), reduction in percent body fat (PRE: 14.44 ± 3.33%; POST: 13.61 ± 3.16%; *p* < 0.05), and increase of WAnT parameters (*p* < 0.05) in the HT group. No changes were observed in the SED group. HIIT resulted in beneficial effects in the increase in blood irisin concentration, physical performance, and reduced fat content. The HIIT may indicate an acceleration of base metabolism. This effect can be utilized in the prevention or treatment of obesity.

## 1. Introduction

High-intensity interval training (HIIT) is a popular form of training among athletes that is used to further improve their level of physical activity from its already elevated state. However, HIIT has also aroused considerable interest among amateurs [1,2]. The basis of HIIT is to perform repeated maximum efforts with alternating rest breaks [3,4]. According to Kimm et al. [5], one of the main advantages of this form of training is its short duration and the variety of exercises, which prevents the sessions from becoming monotonous. Bartlett et al. [1] and Jung et al. [6] suggest that HIIT workouts can be a more enjoyable and attractive form of training compared to moderate-intensity continuous workouts utilized for weight reduction. As reported by Gibala et al. [7] and Gillen et al. [8], this training results in adaptive effects similar to low intensity endurance training along with increase of power and anaerobic capacity. Skally et al. [9] and Siahkouhian et al. [10] confirmed that HIIT training can be an alternative to traditional endurance training to bring beneficial physiological and biochemical changes in both healthy and diseased populations. Boutcher [11] and Shehata [12] reported that HIIT can be used as a weight reduction method, while others have reported improvements in blood lipid concentrations and cardiometabolic profiles among obese individuals [13,14]. Gillen et al. [8], Hood et al. [15], and Hoshino et al. [16] observed an increase in the oxidative potential of skeletal muscles after HIIT training. Rakobowchuk et al. [17] found after HIIT a greater glycogen content at rest, lower lactate (LA) production during the effort, improved lipid oxidation, and increased oxygen uptake. According to Stenman et al. [18], HIIT training also stimulates cognitive processes.

Recent evidence suggests that HIIT can be a time-efficient strategy to promote health in sedentary overweight/obese individuals. This may be contrary to the belief held by some health professionals that training programs at high intensity are not appropriate for optimizing fat oxidation and inducing weight loss in this population [19]. Alkahtani [20] observed improved fat oxidation rate in obese individuals after HIIT training. Other authors have reported an improvement in insulin sensitivity in sedentary patients [21] and increased glucose transporter-4 (GLUT-4) expression in individuals with diabetes [22] or reduction in body mass index (BMI) among obese and overweight individuals [23].

The rising popularity of high intensity training [24] including HIIT among athletes and amateurs is confirmed by the number of training programs available. Among these, the most popular training program is the Tabata protocol [25]. In its original version, the protocol consists of a 4 min workout, involving 10 s of rest for every 20 s of work. In this study, the improvement of aerobic and anaerobic performance was recorded. Authors recorded that the 6-weeks of training using high-intensity intermittent exhaustive exercise improved VO_2max_ by 7 mL·kg^−1^·min^−1^ and the anaerobic capacity by 28%. The spread of HIIT protocols has led to the emergence of various training modifications available on the fitness market [26].

During exercise, skeletal muscles secrete many different biologically active substances called myokines; among them is irisin, a fibronectin type III domain-containing protein 5 (FNDC5). Irisin is a relatively recently discovered myokine called an ‘exercise hormone’ [27] as well as an adipokine released by white adipose tissue [28]. Irisin is a protein consisting of 112 amino acids, released into the bloodstream under the influence of peroxisome proliferator-activated receptor gamma coactivator 1-alpha (PGC-1α) expression after proteolytic separation from FNDC5 [29]. Irisin expression has been primarily reported in skeletal muscles and adipocytes, and has also been expressed in the liver and kidneys [30]. Irisin alters the color of white adipocytes to brown and enhances fat combustion, which suggests that irisin increase may be useful for obesity therapy [31]. In adipose tissue cells, irisin stimulates the expression of uncoupling protein-1 (UCP-1) with PGC-1α and numerous genes of brown fat tissue adipocytes [32]. As a consequence, it contributes to an increase in energy expenditure and thermogenesis, which may result in an improved metabolic profile, increased sensitivity of cells to insulin, and intensified glucose and fat oxidation, especially in people with type 2 diabetes [33]. It also seems that irisin exerts an anti-atherosclerotic and neuroprotective effect [34].

In the literature, contradictory results have been reported related to changes in irisin levels in response to exercise programs. More specifically, changes in irisin concentration were not observed after 45 min of running [35] after 12-weeks of endurance training [36], or after 21-weeks of intensive endurance training [37]. However, these oppose the findings from Kraemer et al. [38] and Huh et al. [39] who reported transient elevations in irisin levels after prolonged moderate aerobic exercise, and after a week training with a sprints series, respectively. It has been also been reported that irisin levels increased significantly after one session of HIIT [40]. Additionally, Archundia et al. [41] observed an increase in irisin level after one HIIT session without changes after aerobic capacity. Given this evidence, we hypothesized that irisin level would increase in response to HIIT training.

Therefore, the aim of this study was to clarify whether 8-week HIIT according to the Tabata protocol would affect irisin secretion, aerobic and anaerobic physical capacity, and body composition in men with low physical activity who participated in recreational activities at a fitness club.

## 2. Material and Methods

### 2.1. Participants

The study involved 25 men randomly classified into two groups. The study group (HT) consisted of men (*n* = 15) with low physical activity who voluntarily participated in HIIT at a fitness club (age: 32.39 ± 6.63 years, weight: 81.01 ± 12.44 kg, height: 176.70 ± 7.44 cm, BMI: 25.75 ± 2.94 kg/m^2^). Taking into account the average % FAT in this group, they were classified into normal weight men [42]. Prior to the study, they reported taking up physical activity only occasionally. For the duration of the 8-week experiment, they limited their physical activity solely to the exercises resulting from participation in HIIT. The initial number of participants in the HT group was twenty, however, within 8-weeks, five of them did not qualify. Two withdrew their participation during the training period because of the too high rigor and intensity, and the next three were excluded because of an absence above 10%. The sedentary group (SED) was comprised of men (*n* = 10) who did not undertake physical activity during the entire study period (age: 25.35 ± 3.28 years, weight: 79.05 ± 9.19 kg, height: 179.83 ± 4.08 cm, BMI: 24.16 ± 2.19 kg/m^2^). Similar to the HT group, the sedentary group did not engage in regular physical activity prior to participation in this study. Exclusion criteria included: smoking cigarettes, diabetes mellitus, thyroid diseases, hypertension, joint pain, and musculoskeletal injuries. All participants declared a very good health status, which was confirmed by medical examination. Study participants were approved to engage in HIIT exercise after written agreement with a physician. On becoming familiar with all the conditions of the study, they provided their written consent to take part in the experiment and were informed that they could withdraw from the study at any stage. The study was approved by and performed in accordance with the recommendation of the Bioethics Committee of the University School of Physical Education in Wroclaw, Poland (date of approval 25/11/15). The studies were conducted in accordance with the Declaration of Helsinki.

### 2.2. Methods

**Figure 1:**
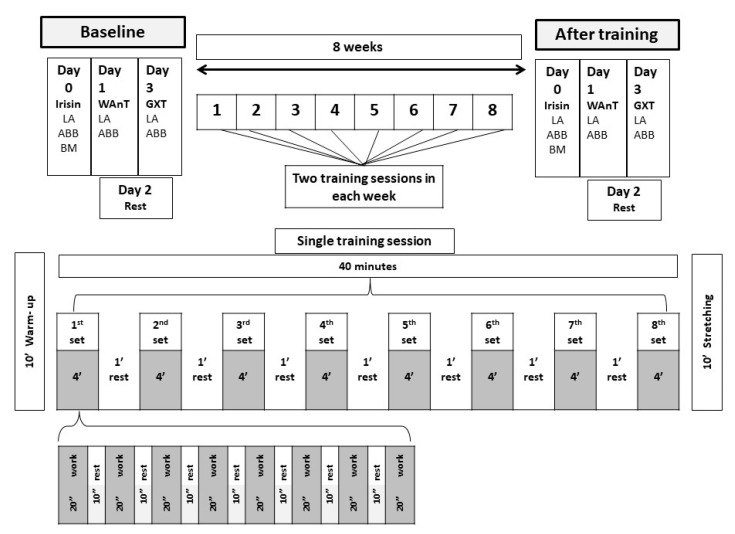
Scheme of the training sessions. LA = Lactate, ABB = Acid–Base Balance, BM = Body mass.

At baseline and after the 8-week training, the participants’ body composition was measured, fasting serum irisin concentration was determined first; on the second day, the anaerobic capacity was established; and on the last day, aerobic power was indicated on the basis of maximal oxygen uptake (VO_2max_) in the order of actions presented in Figure 1.

Body composition analysis was performed in the morning between 8:00 am and 10:00 am, with the use of a BodyMetrix BX 2000 device (IntelaMatrix, Brendwood, CA, USA), in accordance with the manufacturer’s instructions. Prior to testing, participants were instructed to maintain normal hydration, to abstain from the consumption of food and drink 4-h prior to testing, and exercise 12-h before testing. The following body composition parameters were measured: fat content (FAT/kg, % FAT) and lean body mass (LBM). Additionally, the LBM/FAT index was calculated. Each participant’s body mass and standing height were measured to the nearest 0.1 kg and 0.1 cm, respectively, using medical scales (Radwag, Poland).

#### 2.2.1. Aerobic Performance

Aerobic performance was assessed on the basis of VO_2max_. For this purpose, the subjects’ performed graded exercise test (GXT) on a treadmill (SEG-TA7720 treadmill, in SPORT line, Prague, Czech Republic). The test started at a speed of 6 km/h and the speed was increased by 2 km/h every 3 min. Each participant performed the test until failure (i.e., until developing fatigue and discomfort made it difficult to run at the imposed speed). Data were analyzed breath-by-breath and respiratory parameters (VO_2_, VCO_2_, VE) were registered with a Quark b^2^ ergospirometer (Cosmed sri, Rome, Italy). The device was calibrated with atmospheric air and with a gas mixture (5% CO_2_, 16% O_2_, and 79% N_2_). The data of each inhalation and exhalation were averaged in 30 s intervals. During the GXT, maximal oxygen uptake VO_2max_ (mL/min) and VO_2max_ (mL/min/kg), maximal pulmonary ventilation (VE_max_), tidal volume (TV), the time of the test duration, and maximal velocity (V_max_) were assessed. The obtained VO_2max_ was confirmed using established physiological criteria [43]. Heart rate (HR) was measured continuously by telemetry with the use of a POLAR m 400 sport tester (Polar Electro OY, Kempele, Finland). Before and after the GXT, finger capillary blood samples were collected and LA concentrations were determined in 5 min of rest. 

#### 2.2.2. Anaerobic Performance

Anaerobic performance was evaluated on the basis of the Wingate anaerobic test (WAnT), performed on a cycloergometer Monark 894E Peak bike (Monark Exercise AB, Vansbro, Sweden). The Wingate test is a cycle test of anaerobic leg power, conducted over 30 s. This test was designed to measure the maximal anaerobic power and anaerobic capacity, which are extremely essential factors in sports with all-out efforts. Maximal anaerobic power reflects the maximal rate of anaerobic adenosine triphosphate (ATP) synthesis. Anaerobic capacity assessment is the maximal amount of ATP that can be supplied by anaerobic metabolism [44]. The load was individually selected and corresponded to 7.5% of the participant’s body weight. The test was preceded by a 5-minute warm-up with a 50 W load. The following parameters were recorded: maximum power (P_max_–*W; W/kg*), time to obtain P_max_ (T1), time of P_max_ maintenance (T2), mean power (P_mean_), minimal power (P_min_) generated in the last second of test, index of fatigue (IF), total work (TW*–kJ; J/kg*) during the test, and LA concentration measured 5 min after the test.

### 2.3. Biochemical Analysis

At baseline and after the 8-week training, a fasting basilic vein blood sample was collected between 8:00 am and 10:00 am on the day before laboratory testing. The blood was placed in a tube with rapid serum separation granules (Sarstedt, Poland). The blood was then centrifuged, serum was extracted, and the samples were frozen at a temperature of −80 °C. After obtaining the serum from all study participants, the samples were defrosted and irisin concentrations were determined.

In addition to the above fasting sampling, arterialized capillary blood was also drawn at rest (day 0) as well as on test days, before and 5 min after GXT and WAnT, to determine acid–base balance parameters (pH, pO_2_, pCO_2_, BE, HCO_3_^−^) by RAPIDLab 348 analyzer (Siemens Healthcare, Germany).

Serum irisin concentrations were assessed using the enzyme-linked immunosorbent assay (ELISA) with a reagent kit by BioVendor Laboratorni medicina (Brno, Czech Republic), in accordance with the manufacturer’s instructions (intra-assay coefficient of variation: 6.90%, inter-assay coefficient of variation: 9.12%). The lowest detectable irisin concentration in the assay was 1 ng/mL, with an assay range of 0.001–5 μg/mL.

LA concentration in biochemical laboratory conditions was measured by means of the colorimetry method using an Lactate Cuvette Test kit (Hach Lange GmbH, Düsseldorf, Germany) in a Mini Photometer Plus LP20 (Dr. Lange, Germany). The normal range of values was 0.6–0.9 mmol/L. LA concentration during the training in the fitness club was assessed in capillary blood with the enzymatic amperometric method using the Lactate Scout system (RedMed, Warszawa, Poland). The assay range for the method was 0.5–25 mmol/L.

### 2.4. High Intensity Interval Training (HIIT) Training

The training lasted 8-weeks and was performed twice a week (Tuesday and Friday) between the evening hours of 20:00 and 21:00. Overall, 16 training sessions were carried out. Each session lasted 60 min and consisted of a 10-minute warm-up, a 40-minute HIIT training primary portion, and a 10-minute final stretching portion. During the main portion of the session, the participants completed eight exercise series in each training session. Each series lasted 4 min and consisted of eight repetitions of exercises with 20 s of work alternated with 10 s of rest [24]. The main goal of each training session was to perform exercises with maximum intensity. Each series was followed by a 1 min passive break. In each series, the subjects performed different systemic exercises in the following order: Series 1: lower limb muscle exercises (Squats with jumps); Series 2: dorsal muscles exercises (Back Extensions); Series 3: straight abdominal muscle exercises *(Crunches)*; Series 4: chest muscle exercises (Push-ups); Series 5: arm muscle exercises (Triceps Dips); Series 6: abdominal oblique muscle exercises (Side Crunch); Series 7: shoulder girdle muscle exercises (Military press with medicine ball); and Series 8: trapezius muscle exercises (Chin-ups). The final part of the training lasted 10 min and was devoted to calming and stretching exercises. The intensity of each training session was assessed by monitoring heart rate and was averaged for each participant and across entire session. For this purpose, the Polar Team 2 (Kempele, Finland) measuring equipment was used. The energy expenditure was calculated based on the session’s HR of each participants and averaged. Previously, the relation between VO_2_ and respiratory quotient (RER) and HR and VO_2_ was established from the GXT results and energy expenditure calculated. 

Additionally, finger capillary blood samples were collected in order to determine the LA concentrations. These samples were collected 5 min after the main portion of the second training. The training schedule is shown in Figure 1.

### 2.5. Statistical Analysis

The statistical analysis of the obtained results was performed with Statistica 13.0 software (TIBCO, Palo Alto, CA, USA). The sample size (*n* = 25) for two samples and effect size (0.8) resulted in power (0.79), and the study group proportion 0.7/0.3 showed an optimal group sample size of *n* = 24. Normality of distribution was assessed with the Shapiro–Wilk test. The training intensity and LA level was analyzed using the analysis of variance (ANOVA) for repeated measurements in the training sessions and training weeks (intensity/LA × week/set), followed by the Student’s t-test to identify the point of differences between variables. The Student’s t-test was used to evaluate the differences between anthropometric, condition, and LA and irisin variables at baseline and post-intervention. Between-group differences were established with the use of the Mann–Whitney U test. The Pearson’s correlation was used to check the irisin correlation with the other parameters of acid–base balance and both adopted performance tests. The level of *p* < 0.05 was assumed as statistically significant.

## 3. Results

**Figure 2:**
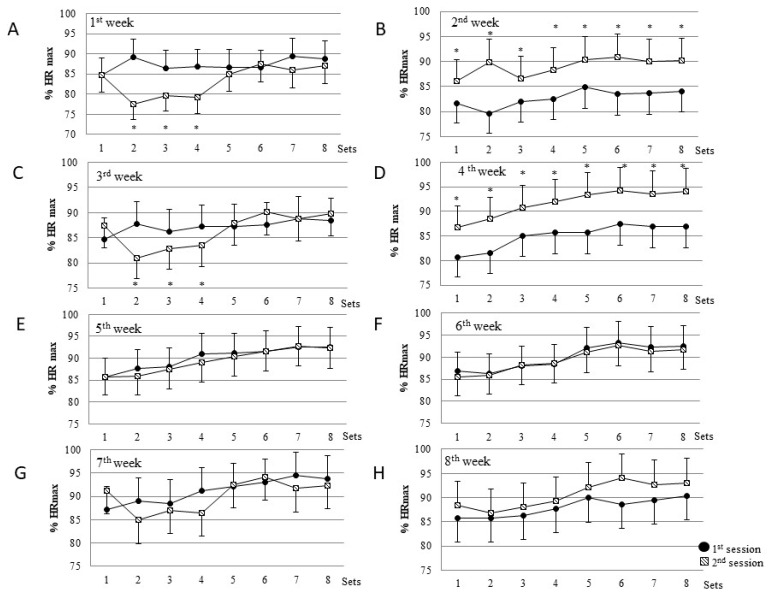
Mean intensity of eight sets in each training session during eight weeks training expressed as % HR_max_. (**A**–**H**) present the intensity of all eight training sets during the first session in the week (black circle) and second session in the week (dotted square) * *p* < 0.05 in comparison to the same sets during the first training session (Student’s t-test). HR = hart rate.

A total of 16 training sessions were performed, with the total working time equaling 80 min each week with associated energy expenditure averaging 882.46 ± 34.25 kcal/week. Training intensity was measured on the basis of heart rate and expressed as % HR_max_ obtained from the GXT. The intensity of work during each training session is shown in Figure 2.

**Figure 3:**
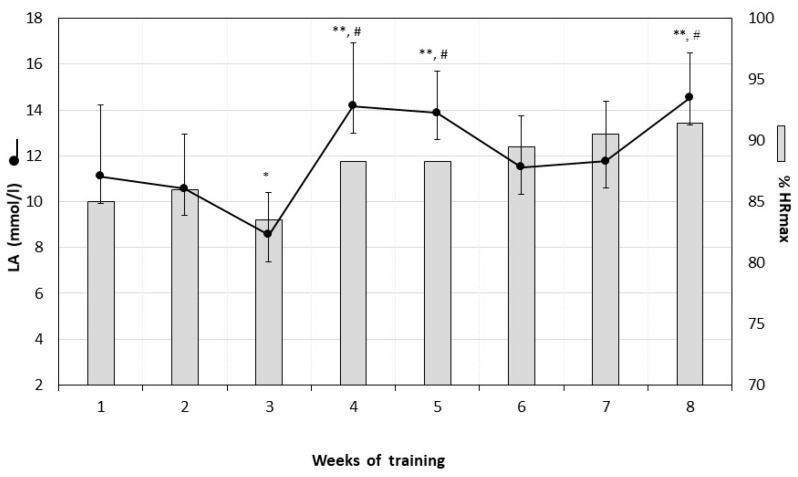
Mean lactate level after two training sessions in each training week and mean % HR_max_ of the two training sessions in each training week. * *p* < 0.05 in comparison to lactate after the first; second; sixth; seventh training weeks; ** *p* < 0.005 in comparison to the third week; # *p* < 0.01 in comparison to the first; second; sixth and seventh weeks of training (Student’s t-test for dependent values). LA = lactate, HR = heart rate.

In the first and third week, the second training session was characterized by a significantly lower intensity of work (Figure 2A,C). In these weeks, the mean HR_max_ of work in series 2, 3, and 4 was lower than 80% HR_max_. In the third training week, the lowest intensity of work was observed, which also corresponded to the lowest LA concentration value after training sessions during the 8-weeks of training (Figure 3). In the second and fourth week, the intensity of the second training session was higher than that of the first session and varied between 87–95% HR_max_ (Figure 2B,D). No significant differences in intensity between the two training sessions were observed after the fifth week of HIIT (Figure 2E–H). The heart rate during both weekly training sessions remained within the range of 85–95% HR_max_.

LA concentration, measured every week after the training session, exceeded the value of 12 mmol/L, with the exception of the third week, in which the lowest LA concentration was recorded after training, along with the lowest work intensity assessed as HR_max_.

After the 8-weeks of HIIT, reduced body weight (*p* = 0.032), reduced FAT (*kg*) (*p* = 0.043), % FAT (*p* = 0.018), and increased LBM (*p* = 0.047) were observed in the HT group (Table 1).

At baseline, a significant difference in FAT between the both groups was noted. The HT group was characterized by having considerably higher fat content, and lower LBM compared to the sedentary group. After the 8-weeks of training, body weight in the HT group decreased and fat content was reduced by 10%. There was also a significant reduction in BMI in the HT group without changes in the SED group (25.75 ± 2.94 kg/m^2^ to 25.24 ± 2.84 kg/m^2^; *p* = 0.035). In addition, LBM and the LBM/FAT index increased in the HT group. After 8-weeks, the SED still presented lower BMI values than those before or after the training in group HT; however, there was no difference in fat content between the two groups. In the SED, % LBM turned out to be significantly lower after 8-weeks compared with the baseline values. When analyzing aerobic capacity before training, we observed that VO_2max_ was significantly lower in the HT group compared to the SED group However, after 8-weeks of HIIT, the aerobic capacity in the HT group significantly improved (Table 1). There was no difference in VO_2max_ between the groups after eight weeks. In the HT group, VO_2max_ increased by 11%, while maximum ventilation and maximum tidal volume were also increased. Additionally, the HT group increased the length in the progressive test performance period. With regard to the WAnT results, a significant improvement in speed and strength abilities among the HT group participants was observed. A significant increase of P_max_ (W and W/kg) and shortening of the time to obtain P_max_ were noted. LA concentration was significantly lower following the test (Table 2).

**Figure 4:**
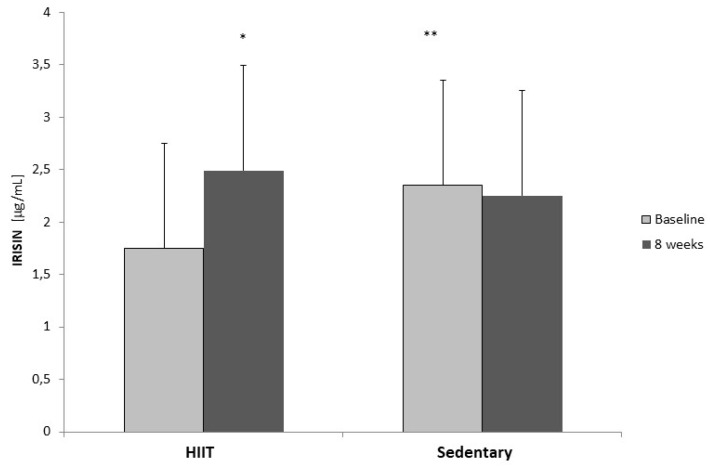
Irisin level in HT and sedentary group at the baseline and after eight weeks; * *p* < 0.05 in comparison to baseline value: ** *p* < 0.05 in comparison to the HT baseline group (Mann–Whitney U-test).

Before training, resting irisin concentration in the HT group was significantly lower than in the SED group (Figure 4). After 8-weeks of training, however, the irisin concentration increased significantly (29.7%) in the HT group and remained unchanged in the SED group. No correlation between irisin concentration and body composition parameters was observed; however there was a relationship between irisin concentration and pO_2_ (r = 0.48; *p* = 0.003) measured in day 0 during the fasting stage.

## 4. Discussion

In this study, improvement in cardiopulmonary performance was observed after 8-weeks of HIIT in the training group of men. In addition, changes in body composition parameters and an increase in blood irisin concentration were shown. Overall, VO_2max_ increased by 11%, fat content was reduced by 10%, and irisin concentration raised by 29%. This translated into increased LBM and LBM/FAT index, lengthened work period during the progressive test under conditions of severe acidosis, and improved speed and power generated by muscles in the Wingate test. This means that the HT participants were able to perform more work in the progressive test compared to their sedentary counterparts. Therefore, we can confirm our hypotheses and recommend this HIIT protocol for men with normal or high fat content in the body mass. According to Roca-Rivada et al. [28], in cases of abnormal BMI, obesity, or overweight, fat tissue can be a significant source of irisin. In this study, the men in the HT group had BMI values higher than normal when compared to the sedentary group at the beginning of the study. Despite this, irisin concentration was lower in this group than in the sedentary group, with BMI values falling within the physiological norm, which may be due to the LBM in both groups. In the sedentary group before training, the LBM and % LBM was higher in comparison to the HT group. After the eight weeks, this parameter in sedentary group did not change while in the HT group, the LBM significantly improved.

The influence of different forms of physical activity on irisin concentration is not clear. In this study, irisin concentration increased by 30% after 8-weeks of training and was correlated with pO_2_. Huh et al. [39] indicated that irisin concentration increased in less than 5 min after high intensity interval exercise, remained high after 1 h, and then returned to the baseline and correlated with exercise intensity. They also reported that baseline irisin levels were lower in old (vs. young) and physically active (vs. sedentary) subjects. Murawska-Cialowicz et al. [45] noted significantly higher irisin values in women compared with men as well as a lower concentration after 3-months of CrossFit training among women only, without significant changes in men. This was accompanied by a reduction in fat content and an increase in LBM in women. The authors believed that perhaps the higher irisin concentration in women before training resulted from a higher % FAT, which was reduced after training. Stengel et al. [46] reported that irisin concentration was higher in obese patients than in normal body weight and anorexic patients. Moreover, in their studies, irisin showed a correlation with fat mass, BMI, and insulin level. As reported by Timmons et al. [47] irisin is not always stimulated by effort. Additionally, older, and not younger, physically active people are characterized by 30% higher expression of the *FNDC5* gene compared to those with a sedentary lifestyle. Kurdiova et al. [48] also did not find that a 3-month workout in overweight and obese individuals increased *FNDC5* expression in skeletal muscles or blood irisin concentration. In contrast to these studies, Düunwald et al. [33] revealed a significant increase in blood irisin concentration in obese individuals with type 2 diabetes subjected to HIIT training.

The heterogeneous direction of changes in irisin concentration after exercise reported in various authors’ studies may be caused by the study procedure and the time of collecting blood samples after effort. In the research presented in this paper, blood was collected 15 min after test completion. In the study by Dascalopoulou et al. [49], an increase in irisin concentration was observed after various types of effort but remained unchanged immediately upon cessation and for as long as 24 h. Similarly, Norheim et al. [36] reported an increase in the hormone concentration immediately after the effort discontinuation. It seems that the season in which the investigation is carried out may also affect the results, as seasonal variation of irisin secretion was found with peaks in winter (January, February) and in summer (July, August) [50].

Relating our results to the values presented in the available literature, one may presume that the changes of resting irisin concentration in the experimental group were influenced by the increase in aerobic capacity levels. It can be assumed that the increase in irisin concentration is stimulated by oxygen deficiency and glycolytic rate during very short intensive exercise. These anaerobic conditions contribute to mitochondrial biogenesis and thus to increased oxygen uptake and fat disposal. The direction of post-workout irisin concentration changes could also confirm the influence of HIIT training on the improvement of oxygen performance and fat tissue reduction and LBM enlargement.

In our study, a significant increase in maximum power and a shorter time to reach it were observed after the end of the training period in the group subjected to the interval training (reduction by 12%). The same direction of change after eight weeks of HIIT was reported by Foster et al. [51]. These changes indicate an improvement in the participants’ phosphagen power, which can result from an increase in LBM as well as improvements in the maximum rate of phosphocreatine (PCr) consumption, the main determinant of phosphagen power. This depends primarily on the composition of muscle fibers and the degree of their adaptation. Burgomaster et al. [52] achieved raised PCr and ATP concentrations after HIIT training. HIIT training according to the Tabata protocol is characterized by high intensity work alternated with short rest breaks, during which the body is not able to fully regenerate before the next repetition of the effort. Due to the short restitution, the workout may resemble continuous training with variable intensity in heart rate responses. This in turn promotes oxygen capacity development and an increase in VO_2max_.

It can be assumed that the increase in irisin concentration may be stimulated in metabolic acidosis conditions and glycolytic rate during very short intensive exercise. These anaerobic conditions contribute to mitochondrial biogenesis and thus to increased oxygen uptake and fat disposal [31]. The direction of post-workout irisin concentration changes could also confirm the influence of HIIT training on the improvement of oxygen performance and fat tissue reduction and LBM enlargement. In our study, the 8-week training resulted in body weight reduction and higher LBM values in the HT group. Trapp et al. [53] recorded a similar reduction in overall body weight during their 8-week interval training program. These changes took place with a simultaneous VO_2max_ increase.

VO_2max_ changes after interval exercises have been reported by numerous authors. Among them, Talanian et al. [54] implemented a program of 10 × 4 min of work/2 min of rest for two weeks. Burgomaster et al. [52] noted improvements in oxygen uptake after only 6-weeks of HIIT. As shown by Wasserman et al. [55], a higher value of oxygen uptake allows for a larger volume of oxygen extraction from inhaled air as a result of the respiratory system adaptation. This change was also observed in our research. The average increase in the ventilation value equaled 7.18 L/min, which significantly contributed to the rise in VO_2max_. Our sedentary group started the program with higher VO_2max_ in L/min (Table 1), which was not different than the slightly older HT group after the intervention, thus we still can anticipate some improvement in the HT group.

In the current study, the training intensity was in the range of 80–95% HR_max_. LA concentration after training, especially in the last training weeks, varied between a range of 10–14 mmol/L. Such intense training provoked the glycolytic rate to lactate and forced the body to adapt.

In our study, higher LA concentrations were observed in subsequent training weeks. This may prove that the participants were able to use the energy from glycolytic transformations for a longer time and were more efficient at removing LA from the muscle cells and releasing it into the blood. Such a change in direction indicates an improvement of the blood buffer capacity and tolerance of metabolic acidosis induced by work, promoting the use of anaerobic glycolysis as the main metabolic pathway. There is evidence that anaerobic conditions induce the glycolytic rate during exercise and HIF-1 (hypoxia inducible factor-1) facilitates the expression of glycolytic genes that encode phosphofructokinase (PFK), a key enzyme in glycolysis. This contributes to an increase in muscle glycolytic capacity [56,57,58,59,60]. Summer matter et al. [61] indicated that interval training increased the number of LA transporters: monocarboxylate transporter-1 (MCT-1) and MCT-4. Research shows that HIF-1 raises the expression of MCT-4, the isoform predominant in glycolytic fibers that facilitates LA removal from these fibers, and MCT-1, the isoform predominant in oxidative fibers, facilitating LA pick-up and disposal [58]. The lower LA concentrations in our study observed after the WAnT in the second post-interval training assessment can be explained by higher PCr resources that prevent the additional glycolytic need. This is different than other research protocol, where Perry et al. [62] observed increased LA concentrations in blood plasma after a 6-week HIIT program. This change indicates a raised tolerance of metabolic acidosis and a greater share of glycolytic transformations in energy supply. These differences might be due to the duration of intervention and participant condition before the intervention. On the other hand, the HR group increased the LA production during the HIIT sessions.

We have attempted to diminish the limitations of this research to the best of our ability. However, we acknowledge the small sample size, which may limit the applicability of our findings. Nonetheless, the results of this research may be utilized for future studies to calculate effects sizes and future sample sizes. In addition, it may be that the Tabata protocol used in this study may be very strenuous for individuals with low physical fitness levels (i.e., overweight or obese); thus, future studies should consider modifying the training protocol to make it more appropriate for individuals enrolled in the study. Finally, our study evaluated male individuals who, although considered overweight, were relatively healthy. Future studies should consider implementing this type of training with women and individuals with different BMI to more properly evaluate the effects of this type of training in these populations. Another limitation might be in the approach to VO_2_ testing, where current recommendations are the use of breathing averaging [63], which was not applied in our study. On the other hand, the main strength of this study is in the high control of the intervention.

## 5. Conclusions

HIIT training significantly affected blood irisin concentration, which likely resulted from the improvement of the participants’ oxygen performance and changes in their body composition, namely the reduction of FAT and increase of LBM. The training was beneficial in improving aerobic and anaerobic capacity, proving that it also has a high application value for improving physical performance. Implementation of a HIIT program in men with low physical activity resulted in improved aerobic and anaerobic performance and higher fat reduction. HIIT increased irisin levels, helping in fat utilization. Our results could be implemented in practice as a form of therapy for individuals with low physical activity, are overweight, and have metabolic disorders. To our knowledge, this is the first study relating the influence of HIIT programing over an 8-week period among low physically active men. Our study showed that HIIT exercise with a Tabata protocol provided significant improvements in physical performance, body composition, and irisin concentration in men. Further research should be conducted to elucidate the effects of HIIT programs among individuals of different sexes and genders as well as among individuals with different BMIs and % FAT, and in a larger population to further evaluate its effectiveness in the prevention and treatment of metabolic disorders.

## Figures and Tables

**Table 1:** Chosen anthropological and physiological parameters at the baseline and after 8-weeks of training.

Groups	HT			Sedentary			HT vs. Sedentary	
Variable	Baseline	8 Weeks	*p*	Baseline	8 Weeks	*p*	Baseline	8 Weeks
Body mass (kg)	81.01 ± 12.44	79.05 ± 9.19	0.032	79.03 ± 12.05	79.70 ± 9.69	0.243	0.342	0.346
LBM (kg)	64.37 ± 9.58	68.62 ± 9.50	0.047	67.34 ± 8.36	67.72 ± 8.69	0.156	0.254	0.432
FAT (kg)	11.64 ± 4.05	10.43 ± 3.61	0.043	10.92 ± 3.42	11.32 ± 3.72	0.046	0.044	0.087
% FAT	14.44 ± 3.33	13.61 ± 3.16	0.018	12.44 ± 3.97	13.00 ± 4.21	0.096	0.009	0.324
% LBM	84.56 ± 3.37	86.39 ± 3.95	0.028	87.55 ± 6.79	72.34 ± 8.5	0.047	0.010	0.012
LBM/FAT (kg)	1.53 ± 0.70	1.85 ± 0.81	0.041	1.66 ± 0.60	1.59 ± 0.57	0.213	0.432	0.047
VO_2max_ (mL/min)	3658.10 ± 487.04	3982.07 ± 575.95	0.028	3962.00 ± 568.19	4001.23 ± 695.33	0.102	0.021	0.134
VO_2max_ (mL/kg/min)	44.86 ± 5.74	50.16 ± 5.80	0.005	47.16 ± 5.01	47.73 ± 5.07	0.231	0.432	0.059
VE_max_ (L/min)	139.52 ± 23.58	146.70 ± 24.68	0.048	151.50 ± 22.35	153.04 ± 25.31	0.212	0.040	0.062
HR_max_ (b/min)	185.30 ± 8.14	186.33 ± 6.89	0.193	190.11 ± 9.65	190.78 ± 7.81	0.342	0.254	0.102
VT (L/min)	2.86 ± 0.55	2.97 ± 0.69	0.124	2.87 ± 0.71	2.72 ± 0.57	0.143	0.110	0.231
V_max_ (km/h)	16.60 ± 2.32	17.11 ± 1.76	0.049	17.71 ± 1.38	17.95 ± 1.65	0.219	0.231	0.232
Time to exhaustion (min)	16.40 ± 2.94	17.31 ± 2.79	0.005	17.34 ± 1.60	16.90 ± 1.69	0.025	0.097	0.058

The values shown as the mean ± SD; Student’s t-test for dependent values between baseline and eight weeks and Student’s t-test for independent value between groups. HIIT = high intensity interval training, LBM = lean body mass, HR = heart rate, VO_2_ = oxygen uptake, V_max_ = maximum velocity, VT = tidal volume, VE = pulmonary ventilation. HT = group performing high intensity interval training.

**Table 2:** Wingate test parameters at baseline and after 8-weeks of training.

Groups	HT			Sedentary			HT vs. Sedentary	
Variables	Baseline	8 Weeks	*p*	Baseline	8 Weeks	*p*	Baseline	8 Weeks
P_max_ (W)	794.2 ± 125.9	825.6 ± 127.4	0.046	840.3 ± 67.9	836.4 ± 68.8	0.043	0.031	0.437
P_max_ (W/kg)	10.23 ± 0.64	11.87 ± 0.67	0.032	10.56 ± 0.66	10.46 ± 0.42	0.324	0.543	0.032
T_1_ (s)	4.84 ± 1.62	3.79 ± 0.93	0.037	4.71 ± 1.47	4.11 ± 0.65	0.049	0.231	0.031
T_2_ (s)	3.45 ± 1.90	3.09 ± 1.12	0.143	3.41 ± 1.77	2.96 ± 0.75	0.132	0.432	0.237
P_end_ (W/kg)	6.51 ± 0.59	6.35 ± 1.55	0.212	6.41 ± 0.55	6.43 ± 0.62	0.345	0.453	0.543
P_mean_ (W/kg)	8.07 ± 0.69	8.09 ± 1.55	0.103	8.33 ± 0.48	8.28 ± 0.43	0.372	0.076	0.067
IF (%)	24.03 ± 4.85	24.12 ± 3.90	0.324	22.72 ± 5.14	23.63 ± 2.17	0.105	0.003	0.049
TW (kJ)	19.03 ± 2.53	18.86 ± 2.64	0.097	19.91 ± 19.54	19.54 ± 1.66	0.432	0.231	0.067
TW (J/kg)	240.14 ± 13.08	240.72 ± 17.08	0.342	252.85 ± 14.64	242.76 ± 16.50	0.041	0.040	0.342
LA (mmol/L)	11.5 ± 1.5	10.11 ± 1.7	0.022	9.3 ± 2.2	9.2 ± 2.0	0.436	0.032	0.012

Values shown as the mean ± SD; Student’s t-test for dependent values between baseline and eight weeks into group, and the Student’s t-test for independent value between groups. LA = lactate, TW = total work, IF = index of fatigue, P = power, T_1_ = time to reach P_max_, T_2_ = time to maintain P_max_. HT = group performing high intensity interval training.
